# The Association of Acute Kidney Injury With Disease Severity and Mortality in COVID-19: A Systematic Review and Meta-Analysis

**DOI:** 10.7759/cureus.13894

**Published:** 2021-03-15

**Authors:** Trishala Menon, Rohit Sharma, Saurabh Kataria, Sundus Sardar, Ramesh Adhikari, Sohaib Tousif, Hira Khan, Sawai Singh Rathore, Romil Singh, Zahoor Ahmed

**Affiliations:** 1 Family Medicine, Wheeling Hospital, Wheeling, USA; 2 Internal Medicine, Hamad Medical Corporation, Doha, QAT; 3 Neurology and Neurocritical Care, University of Missouri Health Care, Columbia, USA; 4 Neurology, West Virginia University, Morgantown, USA; 5 Hospital Medicine, Franciscan Health, Lafayette, USA; 6 Geriatrics, Brown University, Providence, USA; 7 Medicine, Ziauddin University, Karachi, PAK; 8 Internal Medicine, Islamic International Medical College, Rawalpindi, PAK; 9 Internal Medicine, Dr. Sampurnanand Medical College, Jodhpur, IND; 10 Critical Care, Mayo Clinic, Rochester, USA; 11 Internal Medicine, King Edward Medical University, Mayo Hospital, Lahore, PAK

**Keywords:** covid-19, sars-cov-2, acute kidney injury, acute renal failure

## Abstract

Background and objective

The coronavirus disease 2019 (COVID-19) pandemic has become a global healthcare emergency. The severe acute respiratory syndrome coronavirus 2 (SARS-CoV-2), the causative agent of COVID-19, has a wide range of clinical manifestations ranging from subclinical infection to multi-organ failure. In addition to the respiratory system, COVID-19 also adversely affects the kidneys. In this study, we aimed to measure the prevalence of acute kidney injury (AKI) in COVID-19 and its association with the disease severity and mortality in COVID-19 patients.

Materials and methods

We conducted our study by following the Preferred Reporting Items for Systematic Review and Meta-analyses (PRISMA) guidelines. A comprehensive literature search using four databases (PubMed, EMBASE, Google Scholar, and clinicaltrial.gov) was performed. Our initial search returned 2,771 articles. After excluding review articles, duplicates, and non-relevant studies, we included 20 articles that reported an association between COVID-19 and AKI. We subsequently performed a random effect analysis to find the pooled prevalence, pooled odds ratio (OR) estimates, and 95% confidence intervals for severe COVID-19 and mortality outcomes in AKI using Cochrane RevMan (version 5.4) and R programming language (version 4.16-2).

Results

A total of 14,415 patients from various countries were included. Among the 20 cohorts, the median age was 55.8 ±8.39 years (range: 43-72 years), and 43.78% of the subjects were female. Out of a total of 14,415 patients, 3,820 developed AKI with a pooled prevalence of 11% (95% CI: 0.07-0.15; p<0.01; I^2^=98%). AKI was found to have a significant association with severe COVID-19 disease, with a pooled OR of 8.45 (95% CI: 5.56-12.56; p<0.00001; I^2^=0%). AKI was associated with significantly higher mortality in patients with COVID-19 with an OR of 13.52 (95% CI: 5.43-33.67; p<0.00001; I^2^=88%).

Conclusion

AKI manifests as a common COVID-19 complication, and COVID-19 patients with AKI generally have poor outcomes in terms of disease severity and mortality.

## Introduction

The severe acute respiratory syndrome coronavirus 2 (SARS-CoV-2) is the causative agent of coronavirus disease 2019 (COVID-19). The World Health Organization declared a COVID-19 pandemic in December 2019. COVID-19 generally affects the respiratory system, and the patients predominantly manifest fever, myalgia, cough, dyspnea, and flu-like symptoms. The clinical course of the COVID-19 is highly variable, ranging from asymptomatic infection to respiratory failure, multi-organ dysfunction, and mortality. Apart from the respiratory system, COVID-19 is also associated with hazardous effects on other body systems [1,2]. 

Several studies have underlined that older patients and patients with comorbidities are more susceptible to COVID-19, its more serious complications, and progression to severe disease. Recent literature has revealed renal involvement in COVID-19 patients as well [3]. Organ dysfunction such as those involving lungs and kidneys are associated with a high mortality rate [4]. During the SARS-CoV and the Middle East Respiratory Syndrome coronavirus (MERS-CoV) epidemics, a close relationship between coronavirus infection and acute kidney injury (AKI) was observed [4]. A recent study by Chu et al. reported that 5-15% of coronavirus patients tend to develop AKI [5]. Viral entry and replication into the target cells induce systemic inflammatory responses leading to multi-organ dysfunction. AKI in these patients might be due to hypoxemia, dehydration, underlying disease, or adverse effects of administered drugs [6]. 

In clinical practice, COVID-19 has had a significant impact on nephrology. Our study aimed to evaluate the association of AKI with COVID-19 and the outcomes related to the disease severity and prognosis of SARS-CoV-2 patients with AKI.

## Materials and methods

Search strategy and study design

**Figure 1:**
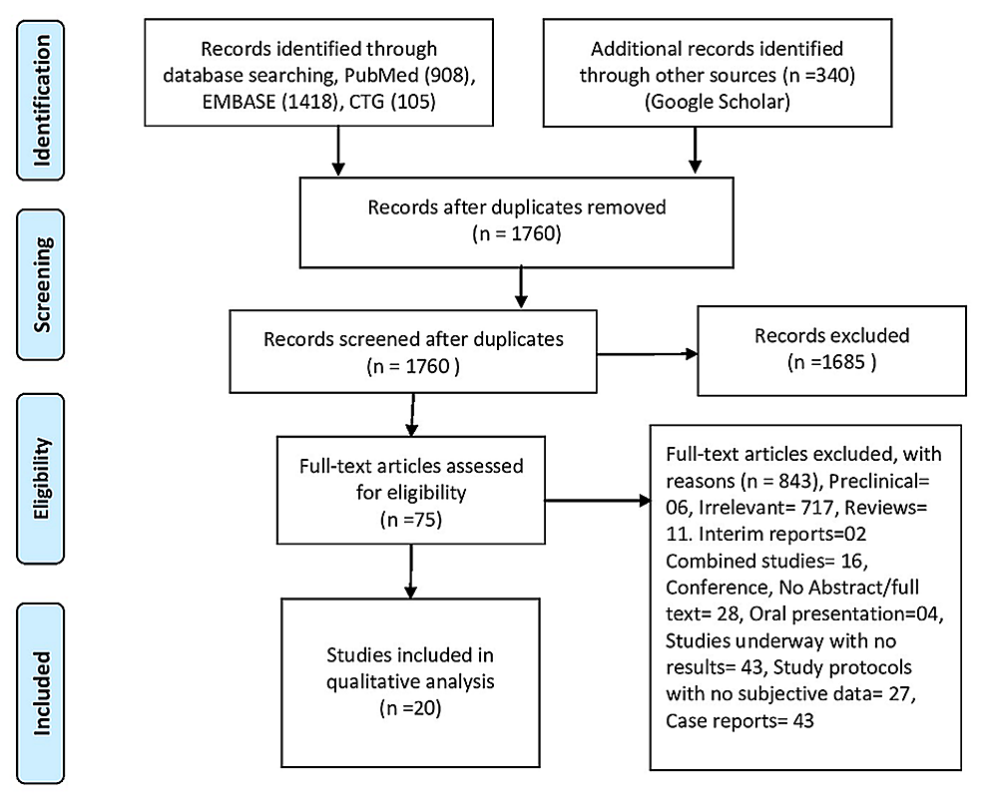
PRISMA flow diagram PRISMA: Preferred Reporting Items for Systematic Review and Meta-analyses.

By following the Preferred Reporting Items for Systematic Review and Meta-analyses (PRISMA) guidelines, we performed a comprehensive literature search using four databases (EMBASE, PubMed, Google Scholar, clinicaltrial.gov) (Figure 1). Our search stratagem relied on the use of the Medical Subject Headings (MeSH) term and keywords for “SARS-CoV-2” and “acute kidney injury” from the date of COVID-19's inception to November 2020. Our initial search returned 2,771 articles. We were left with 75 full-length studies after excluding case reports, animal studies, duplicates, review articles, and irrelevant studies. We finally included 20 articles that reported an association between COVID-19 and AKI after carefully applying the inclusion and exclusion criteria.

Study characteristics and study selection

We exported all studies to EndNote (version 8.0). We screened 669 studies, and 20 articles met our inclusion criteria. Initially, we screened articles by titles and abstract, assessing for relevance. Language restrictions were not imposed. Review articles were excluded. The studies with specialized populations such as cancer and pediatric patients and patients with chronic kidney disease (CKD) and end-stage renal disease (ESRD) were also excluded. We also excluded case reports and randomized clinical trials for drugs. In the beginning, we included full-length articles and then screened each cohort for inclusion criteria. We had prospective and retrospective studies reporting severe COVID-19 and AKI and studies with a more generalized population and detailed extractable AKI-related data. Three authors screened all the articles and assessed full-length studies for inclusion and exclusion criteria. We resolved the disagreements through consensus.

Data extraction and quality assessment

We extracted the clinical information and relevant data using a standard Excel sheet. We reported age, gender ratio, publication year, and country where the study was conducted. We also tabulated outcomes, including AKI incidence (Table 1). In our study, the primary outcomes of interest were severe disease and death. We assessed the quality of studies using the Newcastle-Ottawa Scale (NOS) for quality assessment (Appendices). We also evaluated for the selection bias and small study effects using a random effect funnel plot model.

**Table 1:** Characteristics of included studies AKI: acute kidney injury; COVID-19: coronavirus disease 2019

Author	Study year	Location	Study type	Total number of patients	Median age (years)	Male (%)	AKI in COVID-19 patients
Cai et al. [7]	2020	China	Retrospective, single-center	298	47	50	17
Cao et al. [8]	2020	China	Retrospective, single-center	198	50.1	51	10
Colaneri et al. [9]	2020	Italy	Retrospective, single-center	44	67.5	36.4	2
Feng et al. [10]	2020	China	Prospective, single-center	114	64	62.39	35
Hu et al. [11]	2020	China	Retrospective, single-center	323	61	51.5	17
Huang et al. [12]	2020	China	Prospective, single-center	41	49	73	3
Regina et al. [13]	2020	Switzerland	Observational, single center	200	70	60	30
Wan et al. [14]	2020	China	Retrospective, single-center	135	47	53.3	5
Yan et al. [1]	2020	China	Retrospective, multicenter	2,018	43	56	10
Zhang et al. [15]	2020	China	Retrospective, single-center	221	55	48.9	10
Zhao et al. [16]	2020	China	Retrospective, single-center	91	46	53.8	5
Cao et al. [17]	2020	China	Retrospective, single-center	102	54	52	17
Brill et al. [18]	2020	UK	Retrospective, single-center	450	72	60	80
Chan et al. [19]	2020	USA	Observational, single-center	3,235	64	57	1,406
Hirsch et al. [20]	2020	USA	Retrospective, single-center	5,549	64	60	1,993
Pei et al. [21]	2020	China	Retrospective, single-center	333	57.1	57.1	22
Rubin et al. [22]	2020	France	Retrospective, single-center	71	61.2	77	57
Wang et al. [23]	2020	China	Retrospective, multicenter	138	54.3	57	5
Zhang et al. [3]	2020	China	Retrospective, single-center	663	55.6	48.4	68
Zhou et al. [2]	2020	China	Retrospective, multicenter	191	56	62	28

Statistical analysis

We finally included 20 studies from various countries. We performed a random effect analysis to find the pooled prevalence, pooled odds ratio (OR) estimates, and 95% confidence intervals for severe COVID-19 and mortality outcomes in AKI using the ‘meta’ package by Schwarzer et al. in the R programming language (version 4.16-2), and Cochrane RevMan (version 5.4). The inter-study heterogeneity among the studies was assessed using the Q statistic proposed by Cochrane and the I^2^ index introduced by Higgins and Thompson. We interpreted the values greater than 50% and 70% to be of moderate and high heterogeneity, respectively. The funnel plot was used to explore the publication bias.

## Results

In our study, a total of 2,771 articles were screened, of which 75 full-length studies were further assessed for eligibility, and 20 articles ultimately met the inclusion criteria. These articles involved 14,415 patients from different countries/regions, including Asia, the USA, and Europe. Most studies were done on hospitalized patients. Due to the small cohort's potential overlapping in many studies, we included only 20 unique articles reporting the association of AKI with the disease severity and mortality in COVID-19 patients. Patients who had high serum creatinine after COVID-19 were deemed to have AKI, as per the diagnostic criteria of Kidney Disease: Improving Global outcomes (KGIDO) guidelines. Patients requiring ICU admission, those having severe dyspnea, lung opacities of >50% within one to two days of the disease onset, or oxygen saturation of less than 93 mmHg were considered to have severe COVID-19.

**Figure 2:**
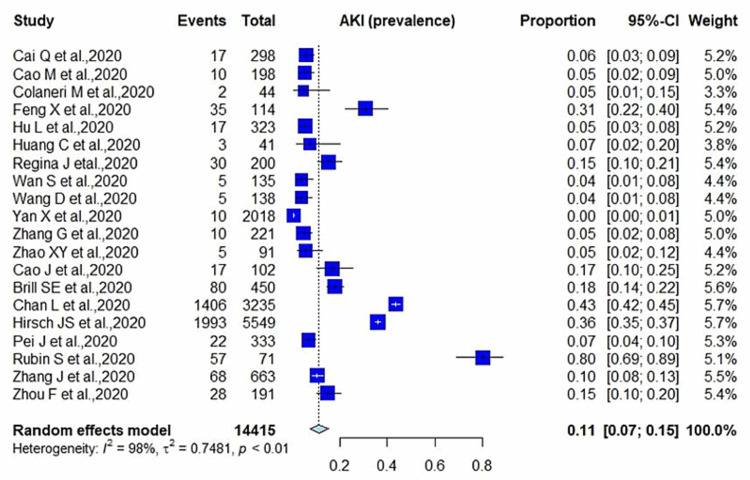
Prevalence of AKI in COVID-19 patients AKI: acute kidney injury; COVID-19: coronavirus disease 2019

Among the 20 cohorts, the median age was 55.8 ±8.39 years (range: 43-72 years), and 43.78% of the patients were female. Out of a total of 14,415 patients, 3,820 developed AKI with a pooled prevalence of 11% (95% CI: 0.07-0.15; p<0.01; I^2^=98%) (Figure 2).

**Figure 3:**
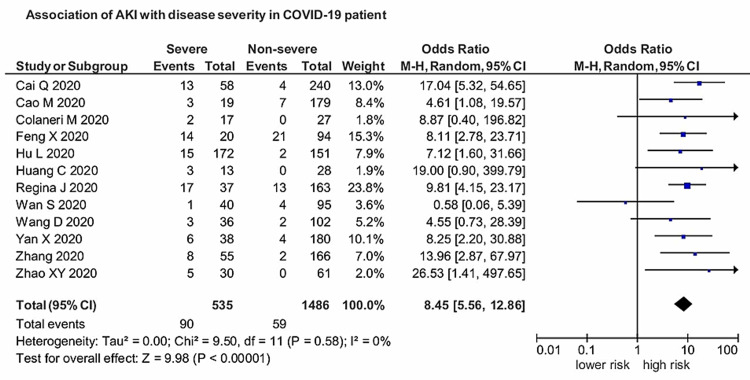
Association of AKI with the severity of COVID-19 AKI: acute kidney injury; COVID-19: coronavirus disease 2019

**Figure 4:**
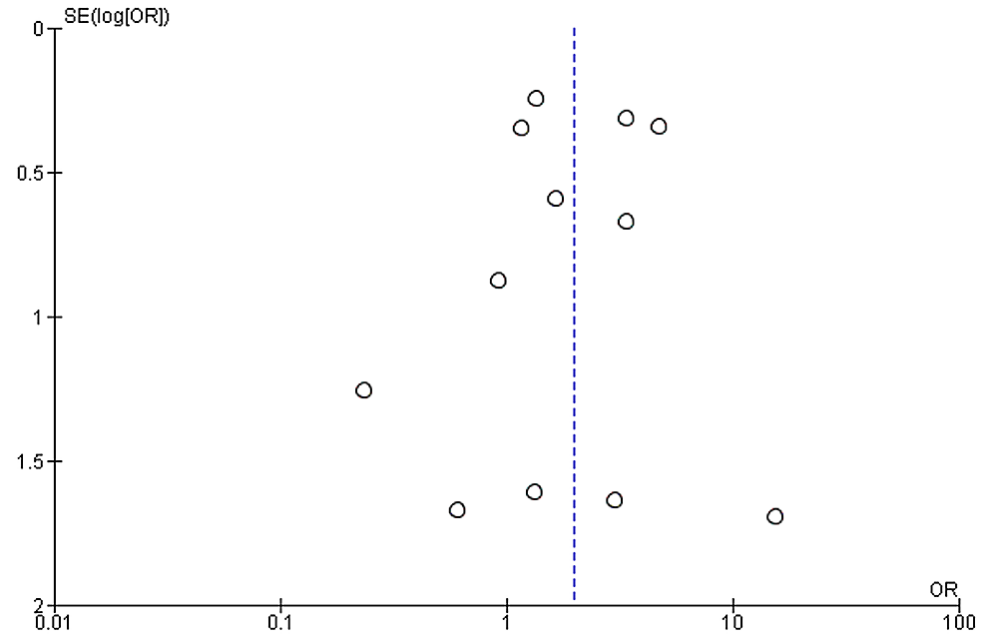
Funnel plot analyzing bias in the association between severe COVID-19 and AKI COVID-19: coronavirus disease 2019; AKI: acute kidney injury

Twelve studies provided sufficient data in their stated outcomes to measure the association of AKI with the severity of COVID-19. AKI was found to have a significant association with severe COVID-19, with a pooled OR of 8.45 (95% CI: 5.56-12.86) compared with patients with the nonsevere disease (Figure 3). The results were significant (p<0.00001), and heterogeneity was negligible (I^2^=0%). The funnel plot showing the association between AKI and severe COVID-19 is presented in Figure 4.

**Figure 5:**
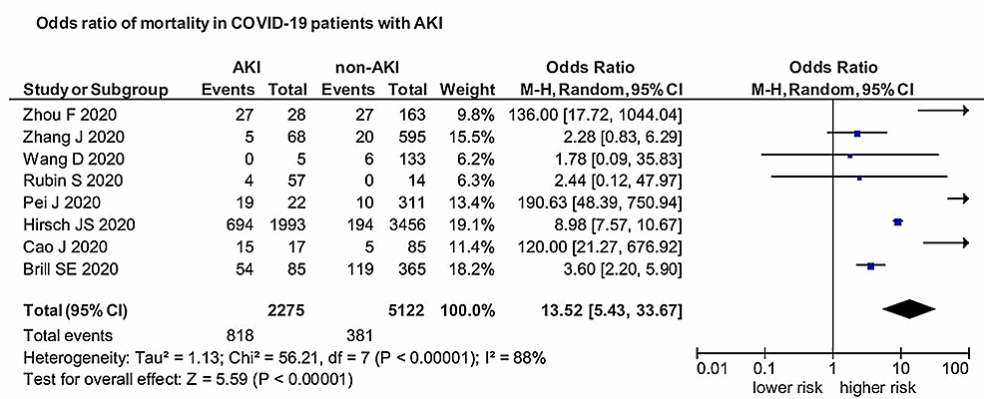
Association between AKI and mortality in COVID-19 patients AKI: acute kidney injury; COVID-19: coronavirus disease 2019

Eight studies provided enough data on mortality in COVID-19 patients with AKI. AKI was associated with significantly higher mortality in patients with COVID-19 with an OR of 13.52 (95% CI: 5.43-33.67). The results were statistically significant (p<0.00001), and heterogeneity was high (I^2^=88%) (Figure 5).

## Discussion

Although respiratory system involvement is the most prevalent feature of SARS-CoV-2, the infection is generally mild; however, severe infection can lead to severe pneumonia, acute respiratory distress syndrome (ARDS), and multi-organ dysfunction, including that affecting the kidneys. Kidney dysfunction is generally mild; however, it is significantly worse in patients with pneumonia. Generally, kidney dysfunction is characterized by hematuria, proteinuria, and elevated serum creatinine and blood urea nitrogen (BUN) levels. BUN and serum creatinine levels, being significant AKI indicators, have been reportedly higher in patients with nonsevere COVID-19 than in patients with pneumonia [5]. This analysis provides evidence of COVID-19 prevalence and association of AKI with the severity and mortality in COVID-19 patients. Our study reported a more potent association between AKI and severe COVID-19 with a pooled prevalence of 11% (95% CI: 0.07-0.15; p<0.01; I^2^=98%). AKI had a strong connection with severe SARS-CoV-2, with a pooled OR of 8.45 (95% CI: 5.56-12.86) compared with those with the nonsevere disease. The results were significant (p<0.00001), and heterogeneity was negligible (I^2^=0%). Regarding the prognosis of COVID-19 patients, AKI was associated with significantly higher mortality with an OR of 13.52 (95% CI: 5.43-33.67; p<0.00001; I^2^=88%). Patients with COVID-19 coupled with AKI have a poor prognosis, and it serves as an independent risk factor for all-cause in-hospital deaths in COVID-19 patients.

Since the emergence of the COVID-19 pandemic, several studies have reported on the prevalence, severity, and prognosis of COVID-19 combined with AKI. A recent study reported a 3% incidence of AKI in hospitalized patients, and this incidence rate increased to 19% when patients with severe disease were included [24]. Moreover, Potere et al. have revealed that the prevalence of AKI was reported to be 6% in hospitalized patients [25]. Lim et al. have reported increased disease severity and mortality in patients with AKI, and the need for ICU care in COVID-19 patients with AKI [26]. Furthermore, in a study by Ali et al., severe AKI was associated with high mortality (relative risk: 3.08; 95% CI: 1.54-6.19) [27]. AKI in COVID-19 is measured as a key marker of severity and has a negative impact on patient survival [2]. We cannot estimate the real burden of AKI in COVID-19 because the serum creatinine value might not reflect the valid preadmission values, and previous serum creatinine values might not be available at that time [21].

Many recent studies have revealed that AKI has a remarkable prevalence in COVID-19 patients, especially those in ICU. The confirmed proportion of AKI in COVID-19 is variable; however, as per the available data, AKI has a prevalence of more than 20% in hospitalized patients and more than 50% in ICU patients [2,17,20,21]. A recent study by Wang et al. reported a significant association between AKI and severe COVID-19 (OR: 11.88; 95% CI: 9.29-15.19) and also reported high mortality in COVID-19 patients (OR: 30.46; 95% CI: 18.33-50-59) [28]. Our study also observed a similar trend, revealing a higher association between AKI and severe SARC-CoV-2 infection (p<0.00001) and increased mortality in AKI patients (p<0.00001). Early detection of AKI and appropriate therapeutic measures are essential to limit morbidity and mortality.

The pathophysiology of AKI in COVID-19 patients is yet to be determined. Several mechanisms have been proposed, including chronic medical conditions such as arterial hypertension, chronic kidney disease, diabetes mellitus, hypovolemic conditions, contrast media, and nephrotoxic drugs. Angiotensin-converting enzyme 2 (ACE2) receptors are the key mediator of viral entry and replication in the host cell. These receptors in multiple organs enable the involvement of different systems, including kidneys and respiratory, nervous, and gastrointestinal systems. In kidneys, ACE2 receptors are expressed in podocytes and apical brush borders of proximal convoluted tubules. ACE2 receptors mediate the viral attachment and entry into the kidney and are responsible for replicating SARS-CoV-2 in the host cells [29]. SARS-CoV-2 also induces an imbalanced activation of the renin-angiotensin-aldosterone system (RAAS), resulting in the downregulation of ACE2 receptors, thereby leading to an increase in angiotensin II. Unprovoked RAAS activation results in a cytokine storm, inflammation, arterial constriction, and fibrosis, and thrombosis at the nephron level [5,24]. Gabarre et al. have proposed that cytopathic activity, cytokine storm, hypoperfusion, and microvascular and intravascular coagulation lead to AKI development in COVID-19 patients [30]. The patients with severe COVID-19 have a high level of inflammatory cytokines, leading to the development of AKI.

Our study has several limitations. We have not included any randomized clinical trials, and all the studies were retrospective or prospective in nature. The individual patient data from each article could not be assessed; thus, we could not make our adjustments. Another limitation is the presence of publication bias, as negative studies were less likely to be published. Also, data on COVID-19 patients is rapidly growing, and many cohorts had a comparatively short follow-up duration and presence of limitations in their detailed description. Moreover, this rapidly emerging data make the retrieval of complete evidence on the subject difficult. We have not included the preprint of the studies, as well as abstracts. AKI after COVID-19 has a significant impact on disease severity and mortality. Our study reported a steady association between AKI and unfavorable outcomes, such as mortality. Given the extent of AKI's impact on COVID-19, emerging literature must provide more comprehensive data on the extent and severity of kidney injury to allow for a proper understanding of the disease prognosis and a comprehensive, resourceful strategy to be adopted during this pandemic.

## Conclusions

There is a growing body of evidence to show that AKI develops in a considerable number of COVID-19 patients, and the condition is significantly associated with adverse outcomes in patients with COVID-19. Thus, it is imperative to exercise extraordinary caution while monitoring the kidney functions of patients regardless of comorbidities and initiating supportive interventions that are surely protective for renal functions in the early stages of SARS-CoV-2 infection. We believe this study will contribute to the physician's essential preparation, which is of utmost significance to managing kidney injury. The only possible way to limit the succeeding AKI in COVID-19 and further progression to more severe stages is to recognize the kidney involvement in COVID-19 at early stages and use preventive and therapeutic measures to reduce morbidity and mortality. Further high-quality data from studies to explore the potential mechanism of the association of AKI with COVID-19 and the underlying cause of mortality are warranted.

## Appendices

**Table 2:** Risk of bias for included studies Risk of bias assessment: each article was given a rating (low=1, medium=2, high=3) according to the NOS. Discrepancies were resolved by a third reviewer if necessary NOS: Newcastle-Ottawa Scale

Author	Reviewer 1	Reviewer 2	Reviewer 3
Cai et al. [7]	2	3	2
Cao et al. [8]	3	3	N/A
Colaneri et al. [9]	3	3	N/A
Feng et al. [10]	3	3	N/A
Hu et al. [11]	3	3	N/A
Huang et al. [12]	3	2	2
Regina et al. [13]	3	3	N/A
Wan et al. [14]	3	3	N/A
Yan et al. [1]	3	3	N/A
Zhang et al. [15]	3	3	N/A
Zhao et al. [16]	3	2	3
Cao et al. [17]	3	3	N/A
Brill et al. [18]	2	2	N/A
Chan et al. [19]	3	3	N/A
Hirsch et al. [20]	3	3	N/A
Pei et al. [21]	3	3	N/A
Rubin et al. [22]	3	3	N/A
Wang et al. [23]	3	3	N/A
Zhang et al. [3]	2	3	2
Zhou et al. [2]	3	3	N/A
